# The Role of Proton-Coupled Amino Acid Transporter 2 (SLC36A2) in Cold-Induced Thermogenesis of Mice

**DOI:** 10.3390/nu15163552

**Published:** 2023-08-11

**Authors:** Hui Shu, Jie Zhang, Dawei Cheng, Xiaorui Zhao, Yue Ma, Chi Zhang, Yong Zhang, Zhihao Jia, Zhiwei Liu

**Affiliations:** Cambridge-Suda Genomic Resource Center, Suzhou Medical College, Soochow University, Suzhou 215123, China; shu_hui@suda.edu.cn (H.S.); jiezhang@suda.edu.cn (J.Z.); mayue@suda.edu.cn (Y.M.); yong.zhang@suda.edu.cn (Y.Z.)

**Keywords:** proton-coupled amino acid transporter, oxygen consumption, brown adipocytes, thermogenesis

## Abstract

Brown adipocytes mainly utilize glucose and fatty acids to produce energy, which play key roles in thermogenesis. Furthermore, brown adipocytes also utilize other substrates, such as amino acids, for energy expenditure in various conditions. Here, we report the new physiological roles of proton-coupled amino acid transporters, SLC36A2 and SLC36A3, on global energy metabolism. The relative mRNA expression levels of both *Slc36a2* and *Slc36a3* were all highest in brown adipose tissue. We then generated global *Slc36a2* and *Slc36a3* knockout mice to investigate their functions in metabolism. Neither loss of *Slc36a2* nor *Slc36a3* affected the body weight and body composition of the mice. *Slc36a2* knockout mice exhibited increased oxygen consumption during the daytime. After cold treatment, inhibition of *Slc36a2* significantly decreased the mass of brown adipose tissue compared to wildtype mice, while it lowered the expression level of *Cpt1a*. Moreover, the serum lipid levels and liver mass were also decreased in *Slc36a2* knockout mice after cold treatment. On the contrary, *Slc36a3* knockout impaired glucose tolerance and up-regulated serum LDL-cholesterol concentration. Thus, SLC36A2 and SLC36A3 play central and different roles in the energy metabolism of the mice.

## 1. Introduction

Adipose tissue (AT) is a highly dynamic organ which accounts for 4–40% of the total body weight in adult humans and plays a key role in systemic energy homeostasis and many other biological processes. The major cell type in the AT, known as adipocytes, are composed of three subtypes: white, beige and brown adipocytes based on morphology, gene expression and function. White adipocytes (WAs) are primarily found within visceral and subcutaneous ATs, where their main function is energy storage in the form of triacylglycerol (TAG) within lipid droplets (LDs). One the otgher hand, brown adipocytes (BAs) reside in brown adipose tissues (BAT) in mammals and have abundantly smaller LDs and dissipate energy through non-shivering thermogenesis [1]. Functional BAs contain numerous and active mitochondria, which express FA/proton symporter protein uncoupling protein-1 (UCP1), that generate heat for adaptive thermogenesis by facilitating proton leak of the mitochondrial inner membrane [2]. In doing so, BAs consume energy and reduce levels of circulating glucose, FAs, and lipoproteins, thereby ameliorating conditions of insulin resistance and hyperlipidemia [3]. Thermogenesis of BAs has been shown to promote energy expenditure and exert anti-obesity and anti-diabetes roles [4,5,6]. Beige adipocytes also have a thermogenic function but are intermingled with white adipocytes within subcutaneous ATs, where WAs and beige adipocytes are interconvertible in response to various physiological and environmental conditions [7]. In addition, all adipocytes can function as endocrine cells to secrete bioactive cytokines called adipokines. For example, “batokines” such as EPDR1, irisin and interleukin-6 (IL-6) secreted by BAs mediate AT remodeling and energy homeostasis under various conditions [8,9]. Thus, the energy dissipation and endocrine functions of BAs can be exploited to overcome the emerging global crisis of obesity and metabolic dysfunction.

Despite glucose and fatty acids, BAs also use amino acids (AAs) as major nutrients during thermogenesis [10]. It has been reported that dietary manipulation of essential amino acids, including leucine, arginine, and glutamine, have significant effects on lipid metabolism and glucose utilization [11,12]. However, the knowledge on the roles of various AAs in thermogenesis is still limited [13]. In cold treated mice, levels of branched-chain AAs (BCAAs, including valine, leucine and isoleucine), as well as alanine, threonine, and tryptophan are elevated in BAT [13]. Glutathione, which is synthesized from glutamic acid, cysteine and glycine, is tremendously reduced in BAT after cold treatment, which enhances mitochondrial reactive oxygen species (ROS) production, and promotes UCP1-dependent respiration [14]. Furthermore, BAT could actively utilize BCAAs in mitochondria through AA transporter SLC25A44 for thermogenesis and it promotes systemic BCAA clearance in mice and humans [15]. Leucine deprivation has also been reported to increase the expression of UCP1 in BAT [16]. Thus, the mechanism underlying AA uptake and mobilization within BAs is very important in the regulation of thermogenesis.

Proton-coupled amino acid transporters (SLC36As, also known as PATs) belong to the transmembrane amino acid symporter family with different expression patterns and substrate selectivity [17]. SLC36A1 and SLC36A2 act as H^+^/AA symporters that differ from the other mammalian AA transporters (mostly known as Na^+^/AA symporters or exchangers) [18]. SLC36A1 is a well-characterized member in the SLC36 family, which is found to be highly expressed at the luminal surface of the small intestine in humans [19]. SLC36A1 has a broad substrate (AA) spectrum and is involved in the absorption of multiple orally-active AA-based drugs as well as derivatives [20,21]. SLC36A2 plays a role in the re-absorption of AAs and other derivatives out of the renal filtrate, and is known to be expressed within the apical membrane from renal proximal [22,23]. Compared to SLC36A1, SLC36A2 preserves narrow substrate specificity (glycine, alanine and proline) [24]. Previous studies have identified SLC36A2 as a specific cell surface marker of BAs, while knockout or overexpression of *Slc36a2* inhibit acidification of lysosome and S6K re-phosphorylation under the starvation condition [25,26]. However, no genetic models have been used to dissect the in vivo function of SLC36A2, especially its involvement in BA thermogenesis. Less is known about SLC36A3 and SLC36A4, except for their function as AA transporters [27,28]. In the present study, we constructed *Slc36a2* and *Slc36a3* global knockout mouse models to explore the potential functions SLC36A2 and SLC36A3 in metabolism.

## 2. Materials and Methods

### 2.1. Animal Usage and Ethics

Mice used in this research were all from a C57BL/6N background and were bred and housed in the animal facility of CAM-SU (Suzhou, China) with free access to acidified water and standard rodent chow food (radiated and autoclaved). Mouse maintenance and experimental use were all under the guidance and supervision based on animal protocols (ZJ-2021-1) approved by the institutional Animal Care & Use Committee of CAM-SU on 24 December 2021.

*Slc36a2* and *Slc36a3* global knockout mice (Knockout First) were made through blastocyst injection of mutant mouse ES cells (Wellcome Trust Sanger Institute). Chimeric mice were then sequenced and the positive insertion ones were bred with wild type C57BL/6N mice to obtain the *Slc36a2/Slc36a3 Tm1a* mice (abbreviated as *Slc36a2/Slc36a3 KO*).

### 2.2. Preadipocyte Isolation and Adipogenic Differentiation In Vitro

Primary WAT SVF preadipocytes were isolated using collagenase digestion and followed by density separation. Briefly, the inguinal white adipose tissue (iWAT) was minced and digested in 1.25 mg mL^−1^ collagenase type I at 37 °C for 40 min. The digestion was terminated with addition of the same volume of DMEM containing 20% FBS, and centrifuged to remove undigested tissues. Cells were then centrifuged at 1700 rpm for 5 min, and the supernatant was removed to obtain SVF preadipocytes in the pellet. The freshly isolated SVF cells were seeded and cultured in growth medium containing DMEM, 20% FBS, 1% penicillin/streptomycin (P/S) at 37 °C with 5% CO_2_ before reaching a confluence of 100%. For adipogenic differentiation, growth medium was replaced by induction medium (IM, 10% FBS, 2.85 mM insulin, 0.3 mM dexamethasone, 1 mM rosiglitazone, and 0.63 mM 3-isobutyl-methylxanthine in DMEM) for 4 days and then differentiated in differentiation medium (DM, 10% FBS, 200 nM insulin and 10 nM T3 in DMEM).

### 2.3. Body Composition Measurement

Body composition (including total body fat, lean mass and fluid) in live animals without anesthesia were recorded using small animal MRI equipment (Minispec LF50 body composition analyzer, Bruker, Billerica, MA, USA). Each animal was placed in a plastic holder designed for mice without sedation or anesthesia. Subsequently, the holder was planted into the measuring space on the side of the MRI system. To guarantee the accuracy of the results, mice were forced to not move in the holder. Each scan took about 2 min.

### 2.4. Indirect Calorimetry

An indirect calorimetry system (Oxymax, Columbus Instruments, Columbus, OH, USA) was used to measure day and night oxygen consumption (VO_2_) and carbon dioxide production (VCO_2_) of the mice. The system was placed in the animal facility of CAM-SU under a strictly controlled temperature (24 °C) and light-dark cycle (light: 8 a.m.–8 p.m.; dark: 8 p.m.–8 a.m.). Each mouse was housed in a chamber with free access to water and food. The whole experiment was performed for 3 days as the first day was for the mice to adapt the chamber. The data presented were all corrected for energy expenditure levels to the body weight of each mouse. Average day (8 a.m.–8 p.m.) and night (8 p.m.–8 a.m.) energy expenditures were the average mean value of all measured data points.

### 2.5. Treadmill

Mice were first trained 5 days before testing at a speed of 5 m/min for 5 min to adapt to the treadmill. Mice were forced to run with an electric shock setting at constant 0.7 mA on a 15% incline. Then on the day of experiment, running the indirect calorimetry program and the treadmill program at the same time. The treadmill program was set as: 5 m/min for 5 min, then increasing the speed at a rate of 2.5 m/min for every 2 min, finally 25 m/min for 4 min. After 25 min, the treadmill program and the indirect calorimetry program was stopped. Then the mice were removed and the treadmill was cleaned with 75% alcohol.

### 2.6. Lipid Measurement in Serum

Blood biochemistry was performed using a Hitachi 7100 clinical chemistry analyzer following the manufacturer’s instructions. Appropriately 500 μL plasma was collected from each mouse, and transferred to a gel tube containing lithium Heparin. Then, 160–200 μL serum was obtained by centrifugation at 5000 rpm using a refrigerated centrifuge set at 4 °C for 15 min. If the volume of serum was insufficient for loading, it was diluted with deionized water to a ratio of 1:2.

### 2.7. Lipid Measurement in Liver

Total TG from liver samples of *Slc36a2* KO or control mice were measured by total TG kit (NJJCBIO, a110-1-1) according to the manufacturer’s protocols.

### 2.8. RNA Extraction and Relative Gene Expression

Total RNA was extracted from adipose tissues using Trizol Reagent (Invitrogen) following the standard protocol from the manufacturer. Absorption ratios of 260/280 nm (~2.0) were measured using a Nanodrop 3000 spectrophotometer. cDNA was then made through reverse transcription using 3 μg of RNA with random primers and M-MLV reverse transcriptase. Real-time PCR was then carried out using the SYBR Green method on a Roche Light-cycler 480 PCR System. Sequences of the gene-specific paired primers were retrieved from PrimerBank and they are listed in Table 1. The relative gene expression levels were calculated using the 2^−ΔΔCT^ method normalized to mouse β-Actin as the internal control.

### 2.9. Statistical Analysis

The two-tailed and unpaired Student’s t test was conducted to calculate the significance of all analyses as presented by mean ± SEM. Statistically significant results were shown by *p* values of <0.05, <0.01 or <0.001.

## 3. Results

### 3.1. Slc36a2 and Slc36a3 Are Highly Expressed in Adipose Tissue and Upregulated during Adipocyte Differentiation

**Figure 1:**
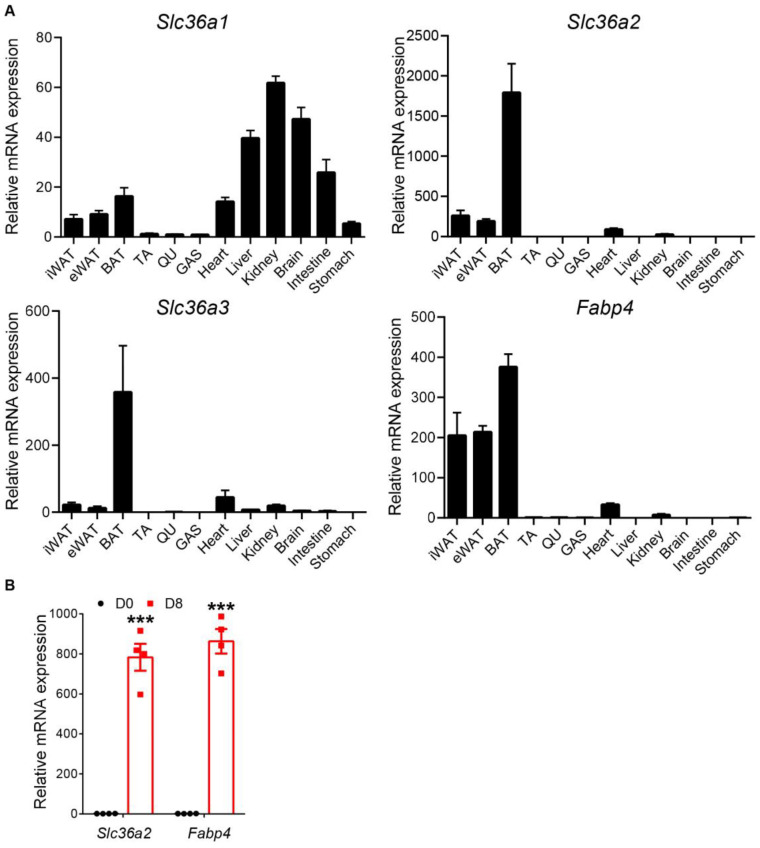
*Slc36a2* and *Slc36a3* are highly expressed in adipose tissue and upregulated during adipogenesis. (**A**) qRT-PCR detection of *Slc36a1*, *Slc36a2*, *Slc36a3* and *Fabp4* expression in different mouse tissues (*n* = 4). (**B**) Relative levels of *Slc36a2* and *Fabp4* at d0 and d8 during adipogenic differentiation of preadipocytes isolated from iWAT (*n* = 4). Data represent mean ± s.e.m. (*t*-test: *** *p* < 0.001).

In order to access the potential roles of SLC36As, we first checked their expression patterns in different tissues. Among the four family members, *Slc36a1* mRNA levels were highest in the kidney, with lower expression levels from the brain and liver (Figure 1A). *Slc36a2* and *Slc36a3* shared similar expression patterns with an adipocyte maker gene *Fabp4* (fatty acid binding protein 4). Their mRNAs were all highest in BAT, followed by lower expression levels in inguinal WAT (iWAT) and epididymal WAT (eWAT) (Figure 1A). The mRNA levels of *Slc36as* were low in all tested skeletal muscle tissues, including tibialis anterior, quadriceps and gastrocnemius (Figure 1A). *Slc36a4* was undetermined in all tested tissues. We next examined whether the expression levels of *Slc36a2* and *Slc36a3* were associated with adipogenesis in vitro. The relative level of *Slc36a2* was significantly increased in differentiated stromal vascular fraction (SVF) preadipocytes isolated from iWAT at D8 compared with undifferentiated cells (Figure 1B). Furthermore, the fold change is comparable to *Fabp4*. However, no *Slc36a3* and *Slc36a4* expressions were detected. These results together indicate that *Slc36a2* and *Slc36a3* may play a role in brown adipocyte, while *Slc36a2* might be involved in the adipogenesis in vitro.

### 3.2. Global Knockout of Slc36a2 Has Minor Effect on Body Composition and Muscle Performance

**Figure 2:**
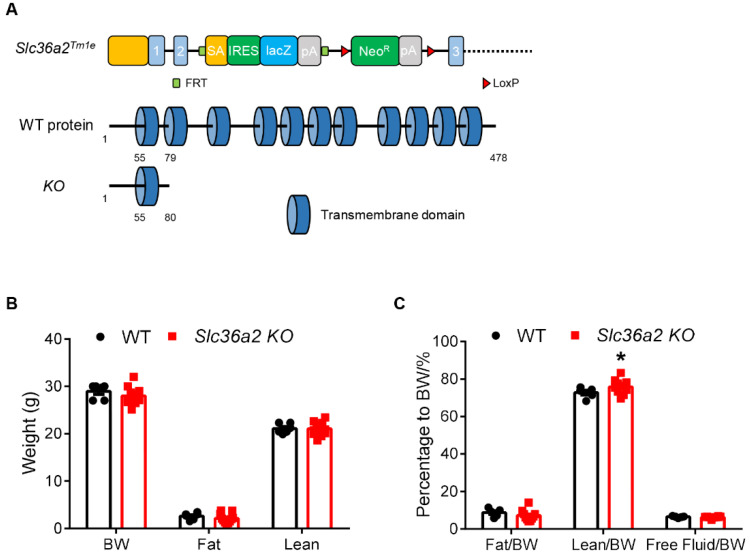
*Slc36a2* knockout does not affect body weight and composition. (**A**) Targeting strategy for global knockout of *Slc36a2*. Upper: Slc36a2 gene structure showing exons (blue boxes). Middle: SLC36A2 protein domain structure with amino acid numbers labeled. Lower: excision of *Slc36a2* results in a premature translational stop, generating a truncated protein containing only part of one transmembrane domain. (**B**) Body weight and body composition of male WT and *Slc36a2* KO mice at 11-week-old, *n* = 6 WT mice and 11 *Slc36a2* KO mice. (**C**) Percentage of body composition to body weight, *n* = 6 WT mice and 11 *Slc36a2* KO mice. Data represent mean ± s.e.m. (*t*-test: * *p* < 0.05).

We next injected a mutant mouse ES cell containing modified *Slc36a2* genome region to obtain *Slc36a2* knockout mice. Homozygous alleles (*Slc36a2* KO) carried the cassette led to early translational termination that generated a truncated peptide, which lost the key transmembrane domains of SLC36A2 (Figure 2A). *Slc36a2* KO was not lethal as the littermates were at expected Mendelian ratio, while the KO individuals were indistinguishable from heterozygous and WT littermates on their morphology. We next measured their body composition at 11-weeks-old. *Slc36a2* KO mice were not different from WT littermates in their body weight, total body fat mass and lean mass (Figure 2B). When calculated the percentage, lean mass ratios of the *Slc36a2* KO mice were slightly increased, but the body fat percentages were unchanged (Figure 2C).

**Figure 3:**
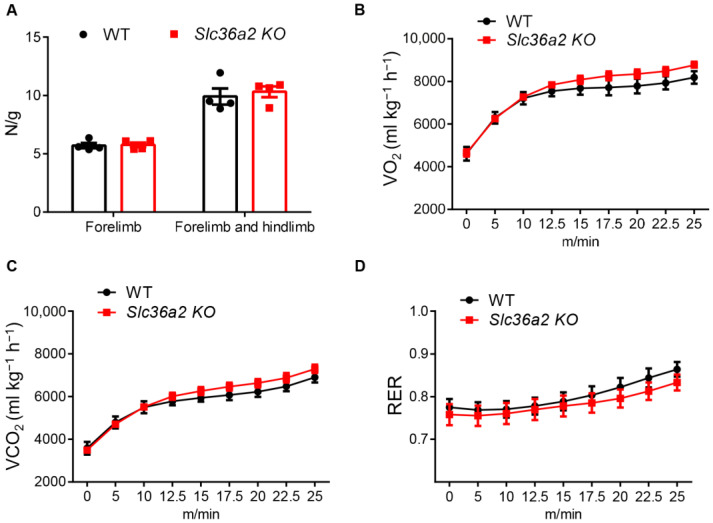
*Slc36a2* knockout does not affect muscle force and exercise performance. (**A**) Grip force of 9-week-old WT and *Slc36a2* KO mice, *n* = 4. (**B**–**D**) O_2_ consumption, CO_2_ production and respiration exchange rate during exercise are measured using a treadmill incorporated with indirect calorimetry. *n* = 6 and 11 male WT and *Slc36a2* KO mice at 12-week-old.

We then inspected the muscle force and exercise performance of WT and *Slc36a2* KO mice. Neither the grip force of the forelimb nor four limbs were different (Figure 3A). We also put the mice on a treadmill plugged into a metabolic chamber to measure their metabolic rates during running. The results showed that WT and *Slc36a2* KO mice had no difference on their oxygen consumption (VO_2_), carbon dioxide production (VCO_2_) and respiration exchange rate (RER) independently of treadmill speed (Figure 3A–C). These results together suggest that knockout of *Slc36a2* has minor effect on body composition and muscle performance of the mice.

### 3.3. Slc36a2 Knockout Elevates the Oxygen Consumption at Daytime

**Figure 4:**
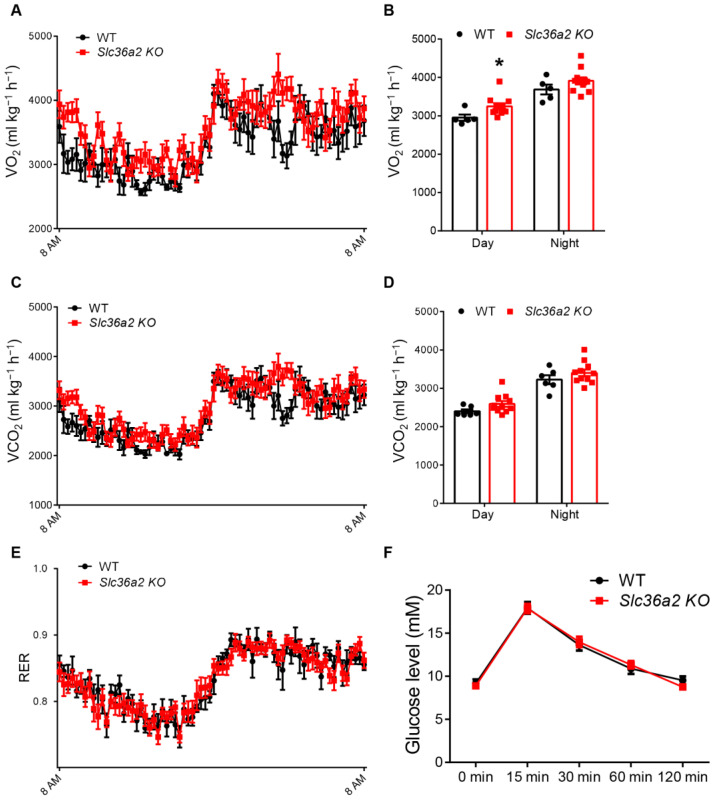
Loss of *Slc36a2* improves O_2_ consumption. (**A**–**E**) O_2_ consumption (**A**,**B**), CO_2_ production (**C**,**D**) and RER (**E**) of 12-week-old male WT and *Slc36a2* KO mice are measured by an indirect calorimetry, *n* = 6 and 11. (**F**) Blood glucose concentrations during the glucose tolerance test (GTT) performed on mice after 12-week-old WT and *Slc36a2* KO mice, *n* = 5 and 10. Data represent mean ± s.e.m. (*t*-test: * *p* < 0.05).

We next examined if the systemic metabolism was affected after *Slc36a2* knockout. Mice lacking *Slc36a2* had higher levels of oxygen consumption than WT mice (Figure 4A,B), especially at around 2 a.m. when mice were actively feeding, and at daytime (Figure 4B). Carbon dioxide production showed a similar trend, but it was not significant (Figure 4C,D). No differences in the RER between WT and *Slc36a2* KO mice were detected (Figure 4E). In addition, the ability for glucose clearance as indicated by the glucose tolerance test (GTT) was also not different between WT and *Slc36a2* KO mice (Figure 4F). These results indicate that *Slc36a2* inhibition elevates oxygen consumption of mice.

### 3.4. Slc36a2 Knockout Mice Had Smaller BAT and Increased Cpt1α Expression after Cold Treatment

**Figure 5:**
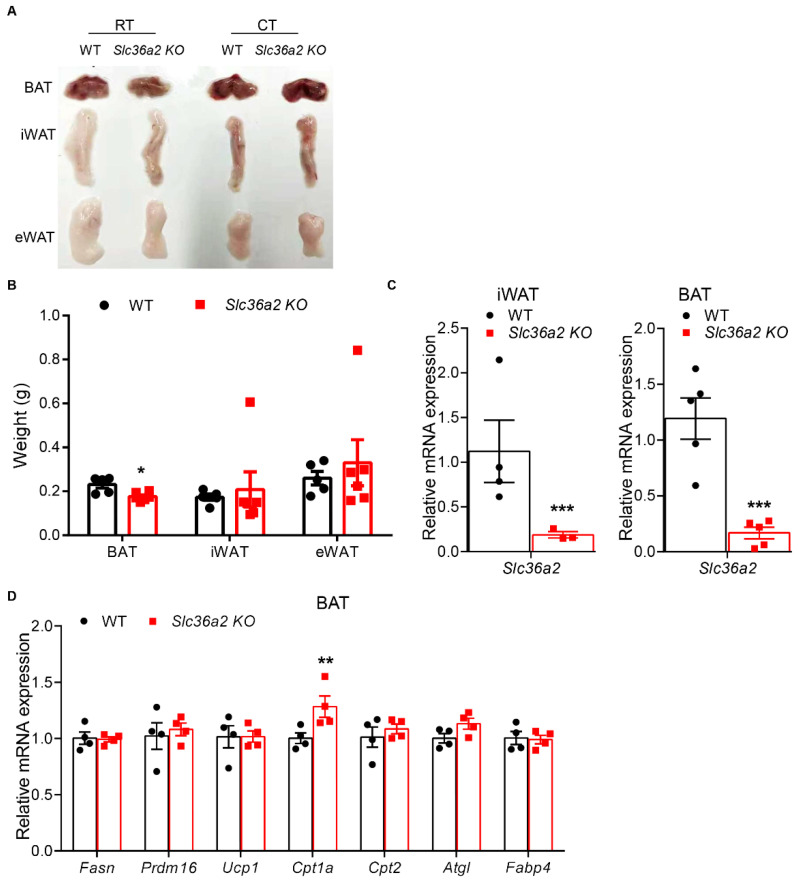
*Slc36a2* knockout reduces BAT mass and upregulates *Cpt1a* after cold. (**A**,**B**) Represent image (**A**) and weights (**B**) of various BAT and WAT (epididymal White Adipose Tissue, eWAT; inguinal White Adipose Tissue, iWAT and anterior subcutaneous White Adipose Tissue, asWAT) depots after 7-day of cold treatment, *n* = 5 and 6 male WT and *Slc36a2* KO mice at 14-week-old. (**C**,**D**) Relative levels of *Slc36a2* (**C**) in iWAT and BAT, and genes involved in TAG synthesis, Adipogenesis, Lipolysis and β-oxidation (**D**) from BAT of WT and *Slc36a2* KO mice after 7-day of cold treatment, *n* = 4. Data represent mean ± s.e.m. (*t*-test: * *p* < 0.05, ** *p* < 0.01, *** *p* < 0.001).

As *Slc36a2* was highly expressed in BAT, we next challenged the mice with cold to further evaluate the role of SLC36A2 during cold-induced thermogenesis. At room temperature, weights of various fat depots, including eWAT, iWAT and BAT, were not different between WT and *Slc36a2* KO mice. The masses of BAT were significantly reduced in *Slc36a2* KO mice than those of control mice after 7 days of cold treatment (Figure 5A,B). However, there were no differences in iWAT and eWAT (Figure 5A,B). As expected, mRNA levels of *Slc36a2* were significantly reduced in iWAT and BAT from the KO mice compared to the WT control (Figure 5C). We next examined expression levels of genes involved in TG synthesis, thermogenesis, β-oxidation, lipolysis and adipogenesis. We only found that the mRNA level of *Cpt1α* was significantly increased from BAT of *Slc36a2* KO than that in control mice (Figure 5D). These results together indicate that loss of *Slc36a2* reduces BAT mass and upregulates *Cpt1α* expression in response to cold.

### 3.5. Loss of Slc36a2 Lowers Lipid Concentrations in Circulation and Liver after Cold Treatment

**Figure 6:**
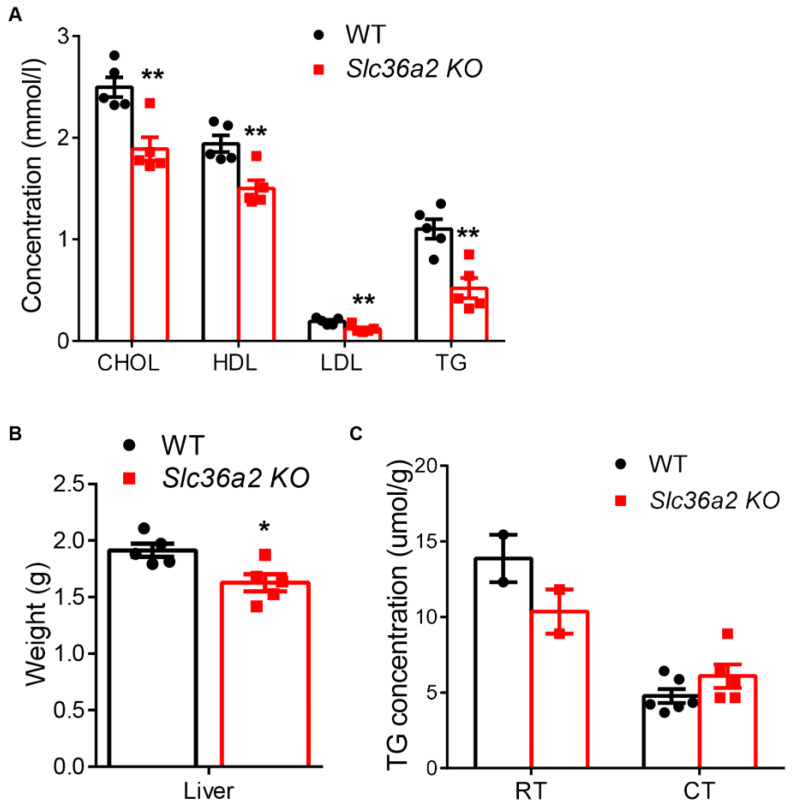
Loss of *Slc36a2* downregulates serum lipids and liver mass. (**A**) Concentrations of cholesterol, HDL, LDL and TG from the serum of WT and *Slc36a2* KO mice after cold treatment, *n* = 5. (**B**) Weights of liver from WT and *Slc36a2* KO mice after cold treatment, *n* = 5. (**C**) TG concentrations in the liver of WT and *Slc36a2* KO mice, *n* = 2 at room temperature (RT) and 5 after cold treatment (CT). Data represent mean ± s.e.m. (*t*-test: * *p* < 0.05, ** *p* < 0.01).

Brown adipocytes dissect glucose and fatty acid for non-shivering thermogenesis and are important in energy homeostasis [29]. The elevated expression of *Cpt1α* in *Slc36a2*-null BAT prompted us to investigate whether loss of *Slc36a2* affected systemic and hepatic lipid metabolism. We performed blood biochemistry analysis using serum samples collected from WT and *Slc36a2* KO mice after cold treatment. Serum cholesterol (total), LDL-cholesterol, HDL-cholesterol and TG levels were all significantly decreased in *Slc36a2* KO mice (Figure 6A). Intriguingly, the liver weight of *Slc36a2* KO mice was also significantly reduced compared to WT control after cold treatment (Figure 6B). In addition, TG content in the liver was also decreased at room temperature, but not after cold treatment (Figure 6C). These data demonstrate that *Slc36a2* loss of function may promote lipid metabolism, especially under cold treatment.

### 3.6. Knockout of Slc36a3 Impairs Glucose Tolerance and Increases Serum Lipid

**Figure 7:**
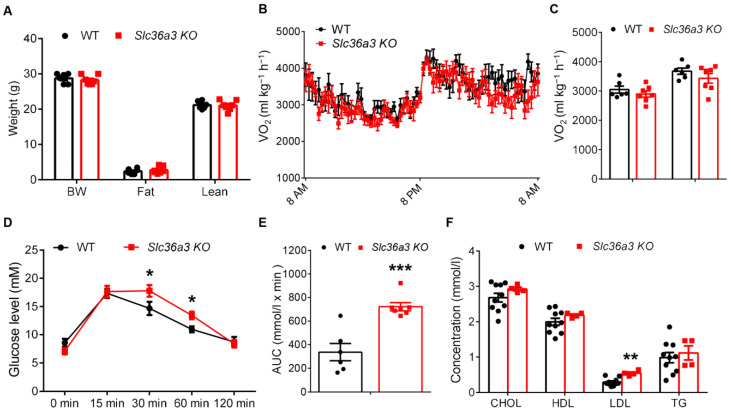
Loss of *Slc36a3* disrupts glucose tolerance and increases serum LDL. (**A**) Body weight and body composition of male WT and *Slc36a3* KO mice at 11-week-old, *n* = 6 WT mice and 7 *Slc36a3* KO mice. (**B**,**C**) O_2_ consumption (**B**) and average day and night O_2_ consumption (**C**) of 12-week-old male WT and *Slc36a3* KO mice are measured by an indirect calorimetry, *n* = 6 and 7, respectively. (**D**) Blood glucose concentrations during the glucose tolerance test (GTT) performed on WT and *Slc36a3* KO mice at 12-week-old. (**E**) Area under curve (AUC) calculated based on data in (**D**), *n* = 6 WT mice and 7 *Slc36a3* KO mice. (**F**) Concentrations of Cholesterol, HDL, LDL and TG from the serum of WT and *Slc36a3* KO mice, *n* = 10 and 4, respectively. Data represent mean ± s.e.m. (*t*-test: * *p* < 0.05, ** *p* < 0.01, *** *p* < 0.001).

Since we found that *Slc36a3* was also highly enriched in BAT, we next examined whether a compensatory *Slc36a3* expression occurred in *Slc36a2* KO BAT. The result showed that the mRNA levels of *Slc36a1* and *Slc36a3* were not changed from iWAT and BAT of *Slc36a2* KO mice compared to WT after cold treatment (Figure S1A,B). Thus, we generated a global *Slc36a3* KO mice using the same strategy to further explore how loss of *Slc36a3* affected energy metabolism. Similar to the *Slc36a2* KO mice, *Slc36a3* deletion was not lethal. We then analyzed the body composition of the mice, and *Slc36a3* KO also did not affect the body composition of the mice (Figure 7A). Both day and night oxygen consumption were not different from *Slc36a3* KO and WT mice (Figure 7B,C), while loss of *Slc36a3* led to impaired glucose clearance as indicated by GTT and the area under curve (AUC) (Figure 7D,E). Moreover, *Slc36a3* KO mice had elevated serum LDL-cholesterol concentration compared to that of WT mice (Figure 7F). Therefore, SLC36A3 may serve a different function compared with SLC36A2, and loss of *Slc36a3* disrupts glucose and lipid metabolism in mice.

## 4. Discussion

Our study demonstrates previously unrevealed roles for SLC36A2 in cold-induced thermogenesis and SLC36A3 in glucose and lipid metabolism. We found that both *Slc36a2* and *Slc36a3* were highly expressed in BAT and that the *Slc36a2* level was increased after adipogenic differentiation in vitro. Loss of either *Slc36a2* or *Slc36a3* has no effect on body weight and body composition. *Slc36a2* knockout increases oxygen consumption of mice during the daytime while *Slc36a3* knockout does not affect the oxygen consumption. Intriguingly, *Slc36a3* KO mice have significantly impaired glucose tolerance which is not observed from *Slc36a2* KO mice. Loss of *Slc36a2* decreases BAT and liver mass, liver TG concentration and blood lipid levels only after cold-treatment, while *Slc36a3* KO increases serum LDL-cholesterol concentration. Further analysis reveals that *Slc36a2* loss of function up-regulates the expression level of *Cpt1a*, which is involved in β-oxidation [30]. Thus, SLC36A2 may serve as an inhibitor of fat oxidation in BAs, while SLC36A3 may play a contrary role to SLC36A2.

SLC36As mediate AA transport and are important for the pharmacokinetic profiles of AA-based drugs by mediating their intestinal and kidney transportation. However, their physiological roles in other metabolic organs in vivo have not been investigated. In particular, SLC36A2 is located on the membrane of BA and may serve as an amino acid sensor to orchestrate the functions of the intracellular organelle [25,26]. Here, we used the genetic *Slc36a2* KO mouse model to access the potential physiological role of SLC36A2 in vivo, especially in BAs after cold treatment. The results indicated that loss of *Slc36a2* improves systemic metabolism, especially lipid metabolism after cold treatment. L-α-amino acids with small aliphatic side chains, such as proline and glycine, are preferred substrates for SLC36A2 [31,32]. It has been reported that proline dehydrogenase (PRODH/POX) affects mitochondrial ROS production when activated [33,34], which subsequently manipulates mitochondrial fatty acid oxidation and oxidative capacity through modulating the flexibility of mitochondria [35,36]. *Slc36a2* inhibition may promote mitochondrial respiration through activation of PRODH. Consistent with our result, knockdown of *Slc36a2* in brown preadipocyte also increases UCP1 level [26]. BAs depends on the H^+^ leak mediated by UCP1 across the inner mitochondrial membrane to generate heat [37,38,39]. SLC36A2 is a pH-dependent, Na^+^-independent and electrogenic AA transporter [40]. In cultured adipocytes, SLC36A2 mediates l-proline uptake in a H^+^-stimulated and Na^+^-independent manner [41]. Thus, inhibition of *Slc36a2* may affect the H^+^ leak and modulates UCP1 activity in BA. Indeed, both *Slc36a2* overexpression and inhibition have been shown to affect lysosomal acidification [26]. However, if the phenotype of *Slc36a2* KO mice was specifically caused by elevated energy expenditure of BA this remains to be investigated by using a BA-specific knockout mouse model. Unfortunately, the third loxP site of the inserted cassette was lost that made it impossible to generate a conditional allele.

Thermogenesis in BAT is involved in the activation of both the sympathetic nervous system and adrenergic signaling from the central nervous system [42,43]. Gamma-aminobutyric acid (GABA) is one of the most well-characterized inhibitory neurotransmitters in the brain that suppresses neuronal excitability [44]. It is reported that in hot ambient air, GABA administration induces lower body temperature in animals [45]. In addition, GABA is reported to be highly activated in obese individuals, which contributes to obese-related BAT and mitochondrial dysfunction. While inhibition of GABA restores BAT function [46]. According to a previous study, SLC36A2 mediates the transport of AA-based derivatives, such as γ-amino butyric acid [32]. It was possible that blockade of SLC36A2 decreased the uptake of γ-amino butyric acid that inhibited the activation of the GABAgenic signaling pathway in BAT, thus promoting the energy expenditure upon cold stimulation.

*Slc36a3* is primarily thought to be an orphan transporter and is only found to be expressed in the testes [17,47]. The physiological roles of *Slc36a3* in vivo and in vitro have not yet been accessed. In the present study, we found its expression was highest in BAT and explored the role of SLC36A3 in vivo by loss of function using a mouse model. The results showed that *Slc36a3* played a role in systemic glucose and lipid metabolism. However, owing to the lack of a specific antibody for SLC36A3, we could not verify the protein localization of SLC36A3 in different tissues. Thus, the tissue specific function of SLC36A3 has not been explored. However, expression patterns of *Slc36a2* and *Slc36a3* mimicked *Fabp4* in vivo, which indicated that SLC36A2 and SLC36A3 may both play roles in AT. Considering the opposite phenotype of the two KO mouse models, we could at least conclude that SLC36A2 and SLC36A3 were indispensable for energy metabolism and there were no compensatory effects in either *Slc36a2* or *Slc36a3* KO mice.

This first in vivo functional characterization of *Slc36a2* and *Slc36a3* assessed the role of SLC36A2 and SLC36A3 by using genetic knockout mouse models, especially in the aspect of metabolism. However, this first in vivo study charactering SLC36As’ function was still limited, especially in terms of technical limitations. First, the current study is mainly based on the results from global KO mice, no conditional alleles have been made to access the tissue-specific roles of SLC36A2 and SLC36A3. Thus, it is impossible now to conclude whether the phenotypes came from BAT. Second, due to the lack of specific antibodies for SLC36A2 and SLC36A3, the endogenous protein levels, distribution and dynamics in various conditions were unable to be characterized. Last, we used KO mice to perform in vivo loss-of-function studies of SLC36A2 and SLC36A3. However, the gain-of-function study through generating overexpression models, especially tissue specific overexpression by transgenic mice or virus injection are still required [48,49]. Thus, future efforts which focus on the tissue specificity of SLC36As will be the next direction.

In conclusion, our results demonstrate that AA transport through various SLC36As is indispensable for BAT and the systemic energy metabolism.

## Figures and Tables

**Table 1:** Primers used in this study.

Primer	Sequence (5′–3′)
Real-time PCR	
*qSlc36a1*	F: ATCAGGAACCTGCGTGTGTT
	R: GTCTTCCATGGAGCCACCAA
*qSlc36a2*	F: GACCAAGAGTGCCAGGAGTC
	R: CCGGTTATGCCCTTGGTCTT
*qSlc36a3*	F: AATGTGCCGCTGCTTAGAGA
	R: TTGAGGAGGCTGTAGACCGA
*qSlc36a4*	F: TGGGATACGGTCCCTCTTGG
	R: GGGCTAGTGTACTGCTGCTC
*qUcp1*	F: AGGCTTCCAGTACCATTAGGT
	R: CTGAGTGAGGCAAAGCTGATTT
*qFasn*	F: GGAGGTGGTGATAGCCGGTAT
	R: TGGGTAATCCATAGAGCCCAG
*qPrdm16*	F: CCACCAGCGAGGACTTCAC
	R: CCACCAGCGAGGACTTCAC
*qFabp4*	F: AAGGTGAAGAGCATCATAACCCT
	R: TCACGCCTTTCATAACACATTCC
*qAtgl*	F: CTGAGAATCACCATTCCCACATC
	R: CACAGCATGTAAGGGGGAGA
*qCpt2*	F: CAGCACAGCATCGTACCCA
	R: TCCCAATGCCGTTCTCAAAAT
*qCpt1α*	F: CTCCGCCTGAGCCATGAAG
	R: CACCAGTGATGATGCCATTCT

## Data Availability

Data is contained within the article or Supplementary Materials.

## Supplementary Materials

The following supporting information can be downloaded at: https://www.mdpi.com/article/10.3390/nu15163552/s1, Figure S1: *Slc36a1* and *Slc36a3* were not changed after *Slc36a2* deletion. (A,B) Relative levels of *Slc36a1* and *Slc36a3* in iWAT (A) and BAT (B) WT and *Slc36a2* KO mice after cold treatment.
