# Supplementary material for: The Role of Proton-Coupled Amino Acid Transporter 2 (SLC36A2) in Cold-Induced Thermogenesis of Mice
Source: Nutrients. 2023 Aug 11;15(16):3552. doi: 10.3390/nu15163552 (PMC10458080; doi:10.3390/nu15163552)
Supplement: Supplementary file 1 [file nutrients-15-03552-s001.zip › nutrients-2523996-supplementary.pdf]

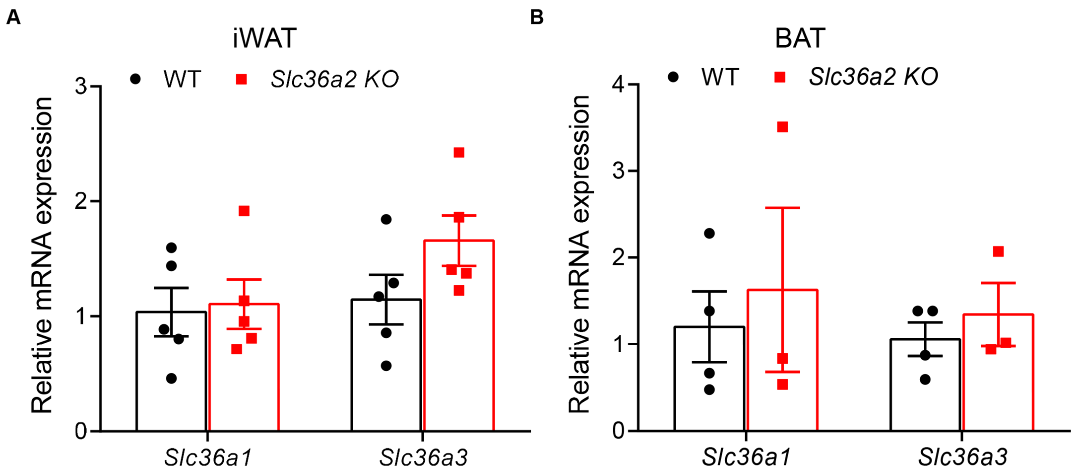

Figure S1. (**A**,**B**) Relative levels of *Slc36a1 and Slc36a3* in iWAT (**A**) and BAT (**B**) WT and *Slc36a2* KO mice after cold-treatment.
